# Fast and Cost-Effective Biochemical Spectrophotometric Analysis of Solution of Insect “Blood” and Body Surface Elution

**DOI:** 10.3390/s18051494

**Published:** 2018-05-09

**Authors:** Aleksandra Łoś, Aneta Strachecka

**Affiliations:** Faculty of Biology, Animal Sciences and Bioeconomy, Institute of Biological Basis of Animal Production, University of Life Sciences in Lublin, Akademicka 13, 20950 Lublin, Poland; los-aleksandra@o2.pl

**Keywords:** hemolymph, immunology, resistance, biochemical parameters, surface proteins, proteolytic system, proteases, protease inhibitors, absorbance, activity

## Abstract

Using insect hemolymph (“blood”) and insect body surface elutions, researchers can perform rapid and cheap biochemical analyses to determine the insect’s immunology status. The authors of this publication describe a detailed methodology for a quick marking of the concentration of total proteins and evaluation of the proteolytic system activity (acid, neutral, and alkaline proteases and protease inhibitors), as well as a methodology for quick “liver” tests in insects: alanine aminotransferase (ALT), aspartate aminotransferase (AST), alkaline phosphatase (ALP), and urea and glucose concentration analyses. The meaning and examples of an interpretation of the results of the presented methodology for biochemical parameter determination are described for the example of honey bees.

## 1. Introduction

Insects are the largest group of animals. They occur in almost all types of environment around the world. There are also cultures of useful insects in which bees, silkworms, and other insects are bred as provender for insectivorous animals and for aesthetic reasons [1]. Arthropods both in the wild and kept under controlled farming conditions may have an elevated or reduced immunity status due to the presence of health-promoting or pathological agents. Positive factors that affect the insects include the use of supplements and drugs, and even antibiotics or natural alkaloids. Among the negative factors that impact the insects, we can identify environmental pollution (e.g., fumes, smog and pesticides, including insecticides), toxins and poisons, pathogenic microorganisms, and parasites [2]. Sick and weakened insects can be a source of infection for other invertebrates; hence, they sometimes cause huge economic losses (it is estimated that the value of pollination by honey bees in one year in the United States is worth about 14.6 billion USD) [3]. They may also cause initially invisible changes that negatively affect other larger organisms, including humans [4]. A cheap and quick analysis that permits the estimation of the health status of insects—e.g., on the basis of proteolytic system activity, “liver” tests, and urea and glucose concentration assays—may be useful as a screening tool and as the first instrument for assessing the positive/negative effects of various substances on the organism. An additional advantage and argument for conducting screening biochemical tests on invertebrates is that they are often treated as lower-level animals; thus, the research using them does not require applying for legal permits and is not restricted in most countries.

## 2. Materials

Even until now, it has been a very common practice to carry out biochemical tests on homogenates made from entire insects [5]. This approach, although still present, is outdated and has many disadvantages. Insects, despite having an open circulatory system, are complex organisms in which we can distinguish tissues, organs, and complex organ systems corresponding to animals of higher orders, e.g., the hemolymph is similar to vertebrate blood [6], and the fat body is partly similar to vertebrate liver [7]. When we biochemically analyze homogenates, confusing results are obtained that are not appropriate for adequate interpretation as a consequence of mutually preclusive reactions in different parts of the insect body. Therefore, many scientists in independent centers use various techniques and methods for obtaining insect tissues for research [6,8,9]. The authors of this manuscript briefly present the most important methods of hemolymph collection, which they consider the most suitable tissue for reliable and conclusive analyses. Additionally, the authors describe a methodology of washing out proteins and other substances from the insect body, as it may be the only possible way to obtain research material from small species or complementary material to the hemolymph. Such elution is also the best material to define the environmental impact [10].

### 2.1. Hemolymph Collection

**Figure 1:**
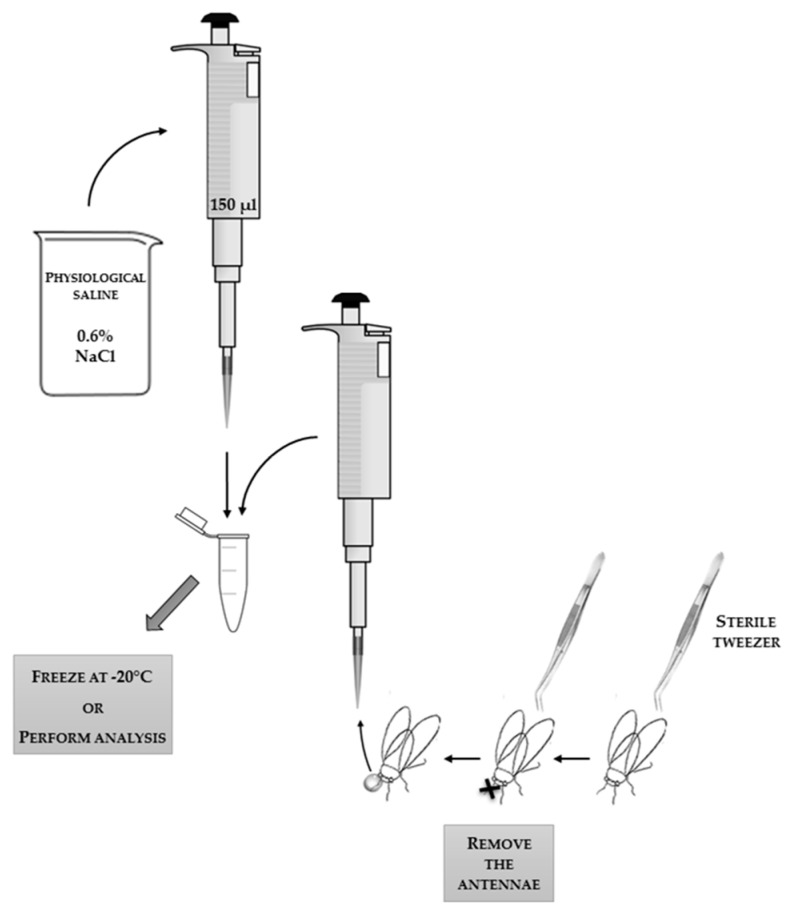
The scheme of the technique of collecting hemolymph flowing out of the insect’s head after detachment of the antennae. The applied tools and reagents, along with the used volumes, are presented in the scheme.

**Figure 2:**
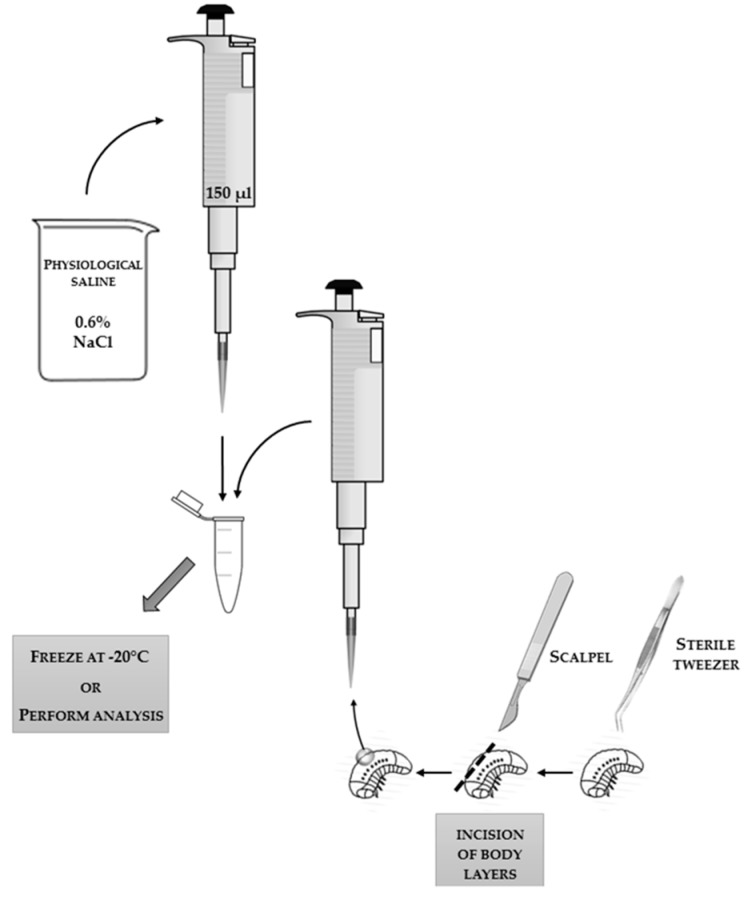
The scheme of the technique of collecting hemolymph flowing out of the insect’s body after incision of the insect’s body surface with a scalpel. The applied tools and reagents, along with the used volumes, are presented in the scheme.

**Figure 3:**
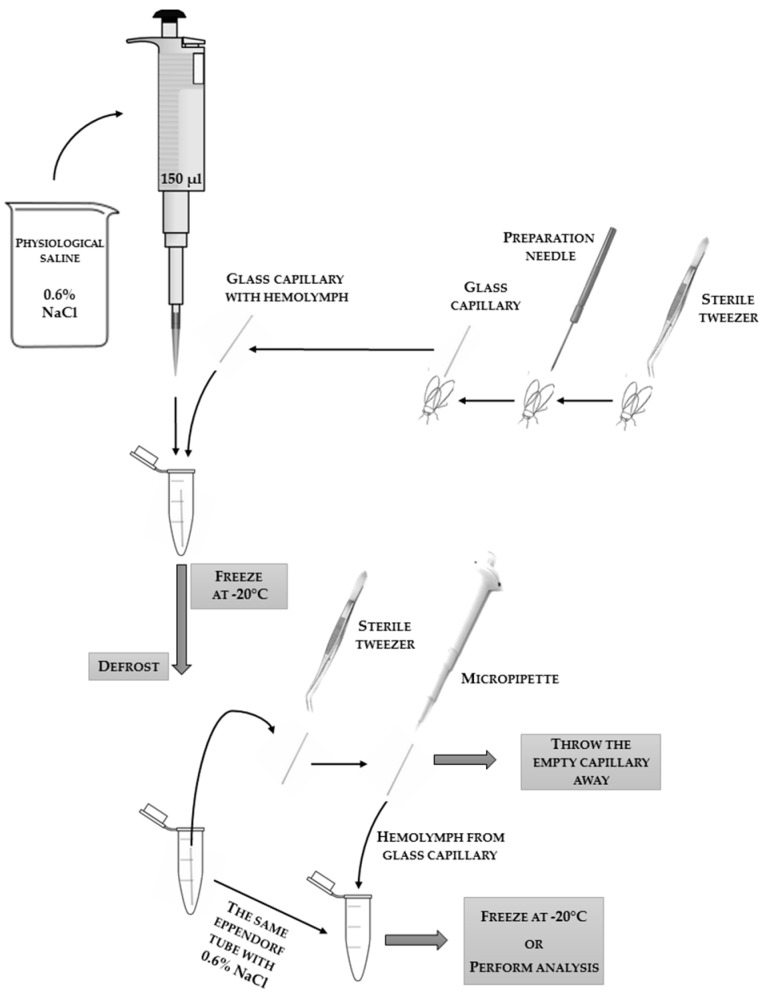
The scheme of the technique of collecting hemolymph into a glass capillary (e.g., 10 μL; the “end-to-end” type; without anticoagulant; Medlab Products) after puncturing the insect’s body (abdomen) with a sterile preparation needle. The applied tools and reagents, along with the used volumes, are presented in the scheme. The bottom part of the scheme shows the steps of blowing out the hemolymph from the glass capillary in order to suspend it in physiological saline to conduct analyses.

**Figure 4:**
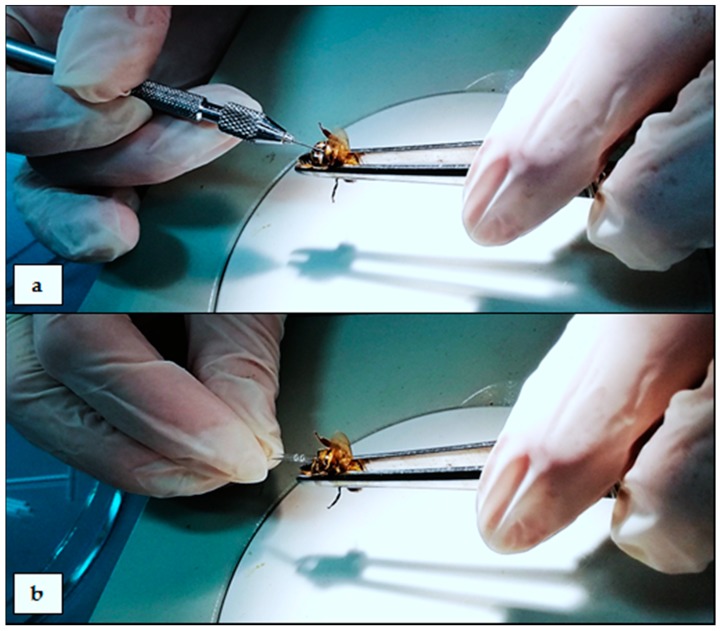
A demonstrative photo of the technique of collecting hemolymph into a glass capillary by puncturing the insect’s body: (**a**) puncturing the venous sinus in the abdomen of the honey bee (*Apis mellifera*) with a preparation needle; (**b**) the hemolymph of a honey bee is spontaneously floating inside the glass capillary placed in the abdominal venous sinus after puncturing it with a preparation needle.

The most clear-cut analyses can be carried out on the hemolymph (“blood”) of insects suspended in 0.6% physiological saline (NaCl) for insects. Methods for obtaining hemolymph are compared in Table 1, and collection schemes are presented in Figure 1, Figure 2 and Figure 3, with an additional demonstrative photo in Figure 4.

### 2.2. Elution from the Insect’s Body Surface

If the insect integumentary system is intact, we can wash all of the substances from the surface into the solution, and we can analyze the obtained elution. At this point, it is worth noting that all of the hemolymph collection methods violate the body surface and disqualify the insect from an analysis of body surface elution (due to the contamination of the body surface with substances from the interior). The immune system of some insects covers the surface of the body; the production of immune proteins on the outside keeps microorganisms from penetrating inwards [11,12,13]. The body surface samples of some insect species could be affected by the metobolic products of surface-residing microorganisms, and results obtained from their analysis are not apropiate for interpretation.

**Figure 5:**
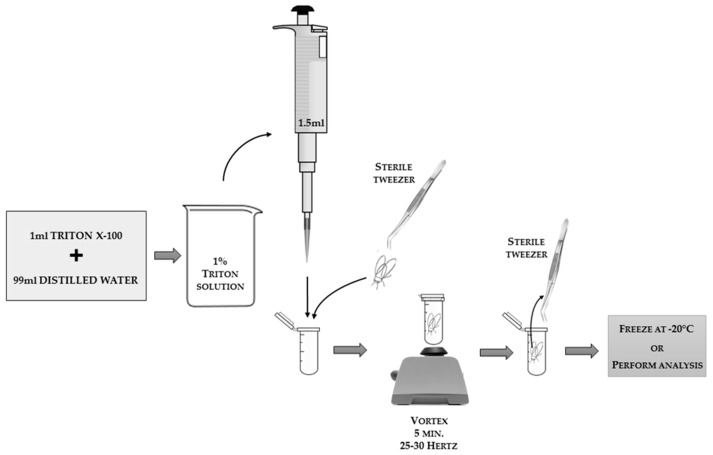
The scheme of the technique for obtaining insect body surface elution. The applied tools and reagents, along with suggested volumes, are presented in the scheme.

As a neutral detergent that effectively washes substances from the body surface, the 1% solution of Triton X-100 (or another detergent with similar properties) is suitable. The insect inside the Eppendorf tube should be poured with the detergent solution in such a way so as to surround the whole body of the insect (e.g., in the case of honey bees, 1.5 mL of solution is an appropriate volume; the volume for smaller or larger insects should be adjusted as follows: solution volume [mL] = insect weight [mg] × 1.5 [mL]/100 [mg]). The scheme of the technique for obtaining body surface elution is presented in Figure 5.

## 3. Methods

### 3.1. Total Protein Concentration

**Figure 6:**
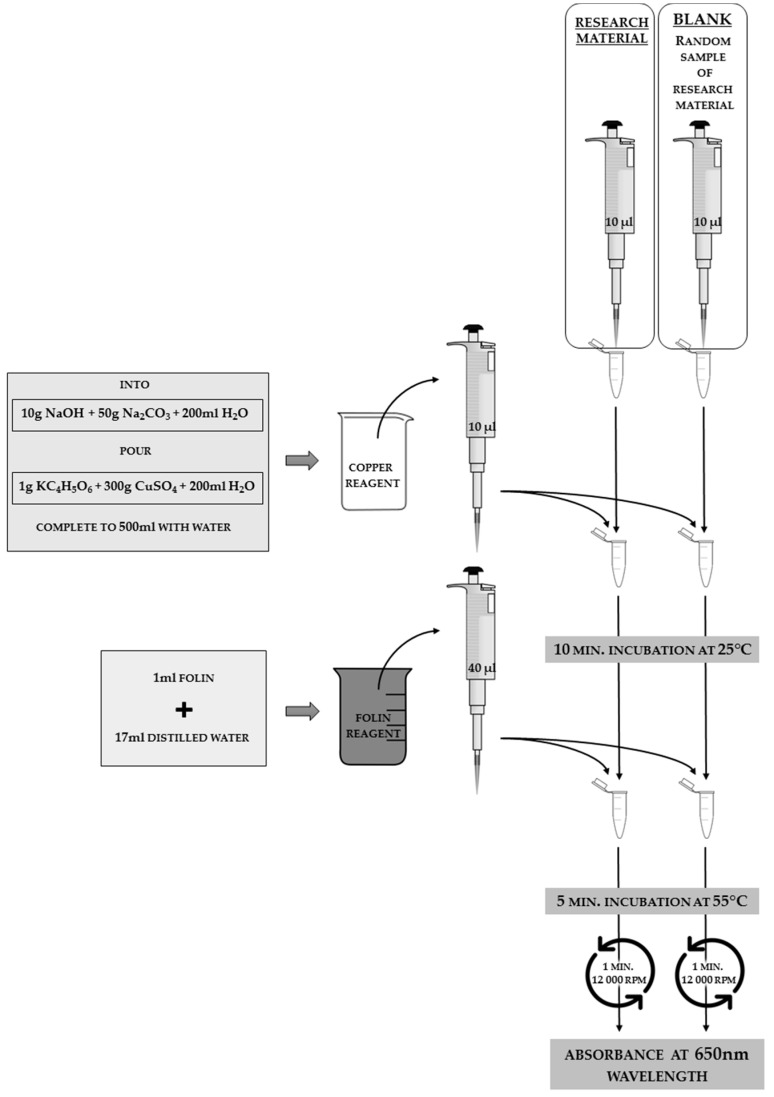
Methodology for analyzing the total protein concentration in the hemolymph solution or in the insect body surface elution. The applied tools and reagents, along with the used volumes, are presented in the scheme. On the left side of the scheme, the exact composition of the copper reagent is presented, as well as the dilution method of the commercially purchased Folin reagent (e.g., Sigma-Aldrich, Folin and Ciocalteu’s phenol reagent), which should be kept in a beaker unexposed to light.

Strachecka adjusted the Lowry method modified by Schacterle and Pollack [14] in order to verify the total protein concentrations of hemolymph solutions or body surface elutions. The consecutive steps involved in the analysis, the substances necessary for the reaction to occur, and the method of handling the sample in order to obtain the solution to be analyzed for absorbance are shown in Figure 6. The value of protein concentration in the sample is necessary for further analysis of the proteolytic system.

The total protein concentration value should not be interpreted as the proper result, because the number of proteins in the solution depends on the individual characteristics of the insect and the circumstances of collecting the biological material (e.g., an insect with no access to water will have a small volume of hemolymph in the body, and therefore only a small volume of hemolymph can be obtained; thus we get a low concentration of proteins, which has nothing to do with the insect’s immunology status). Nevertheless, the obtained value may provide guidance for further proteolytic system interpretation.

It takes approximetely 1.5 h to determine the total protein concentration for 100 samples (this is highly dependable on the laboratory equipment and the spectrophotometer model). The cost of analysis for 100 samples is about $100.

#### Calculation of the Total Protein Concentration

In order to calculate the concentration of the total protein content in the sample, the absorbance value needs to be inserted into the formula, as follows:

(1) $$\text{T}\;\mathrm{OTAL}\;\mathrm{PROTEIN}\;\mathrm{CONCENTRATION}=\frac{\begin{array}{c} SAMPLE\\ ABSORBANCE \end{array}}{FACTOR}\;\left[\frac{\mu g}{\mathrm{mL}}\right]÷1000=\;total\;protein\;concentration\left[\frac{\mathrm{mg}}{\mathrm{mL}}\right]$$

where the applied factor is estimated from the standard curve for albumin.

This total protein concentration result [mg/mL] obtained for a particular sample should be substituted in the calculation formula for the activity of proteases and proteases inhibitors in order to calculate the value for the same particular sample obtained from the same individual insect.

### 3.2. Proteolytic System

#### 3.2.1. Protease Activity Analyses

**Figure 7:**
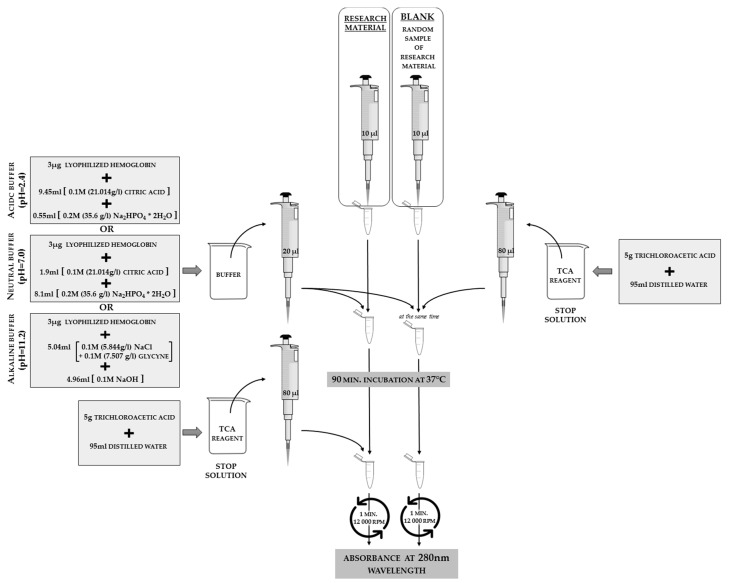
The schemes for acidic (2.4 pH), neutral (7.0 pH), and alkaline (11.2 pH) protease activity analyses in hemolymph solutions or insect body surface elutions. In order to calculate the values of the protease activities from the absorbance results, it is necessary to substitute the blank absorbance into the calculation formula. This step indicates the results for a sample in which all of the reagents are present, but no reaction occurred (stop solution is added at the beginning of the procedure, as shown on the right part of the scheme). The phrase “random sample” refers to the case when we analyze a larger number of samples in one study; we may prepare only one blank for all of the samples that are using the same set of reagents on the same day.

Strachecka modified the Anson methodology [15] in order to verify insect proteolytic system activity. Protease activities are assayed in an acidic, neutral, and alkaline buffer environment. Buffers of defined pH in the range between 2.2–12.8, separated by 0.2 intervals, should be prepared in accordance with Table 2, and checked with a pH meter. Three µg of lyophilized hemoglobin should be added to 10 mL of each buffer. The next steps are the same as those shown in Figure 7. The optimal pH values have been selected in which the enzymes revealed the highest proteolytic activity [12,16,17]. Subsequent analyses are performed at these pHs. The scheme of protease activity analysis is shown in Figure 7.

It takes approximately 2.5 h to determine the protease activity analyses for 100 samples and one pH value (this is highly dependable on the laboratory equipment and the spectrophotometer model). The cost of analysis for 100 samples is about $150.

#### 3.2.2. Protease Activity Level Calculation

In order to calculate the protease activity levels, the absorbance values need to be inserted into the formula, as follows:

(2) $$\text{P}\;\mathrm{ROTEASE}\;\mathrm{ACTIVITY}=\frac{\left(SAMPLE\;ABSORBANCE-BLANK\;ABSORBANCE\right)}{total\;protein\;concentration\left[\frac{\mathrm{mg}}{\mathrm{ml}}\right]\times TOTAL\;INCUBATION\;TIME\left[\min\right]}\left[\frac{U}{\mathrm{ml}}\right]$$

where the total protein concentration is the value obtned in the previous analysis for the same sample and total incubation time is equal to 90 min, according to the methodology from Figure 7.

#### 3.2.3. Protease Inhibitor Activity Analyses

**Figure 8:**
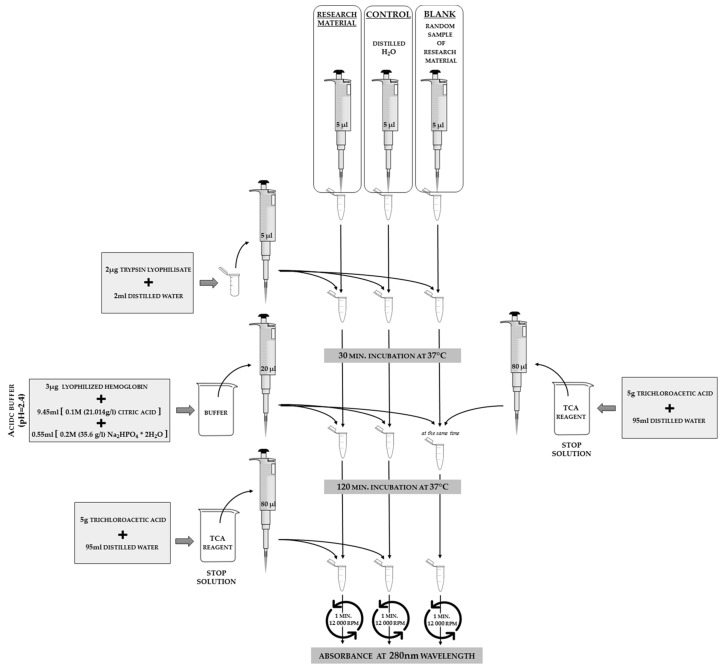
The scheme for acidic (2.4 pH) protease inhibitor activity analysis in hemolymph solutions or insect body surface elutions. In order to calculate the values of the protease inhibitor activities from the absorbance results, it is necessary to substitute the control sample absorbance in the calculation formula in order to determine the results when all the reagents are present but no reaction could occur because there was no biological material. It is also necessary to substitute the blank sample absorbance where the biological material was used, but no reaction occurred (the stop solution is added at the beginning of the procedure, as shown on the right part of the scheme). The phrase “random sample” refers to the case when we analyze a larger number of samples in one study; we may prepare only one control sample and only one blank for all of the samples that use the same set of reagents on the same day.

**Figure 9:**
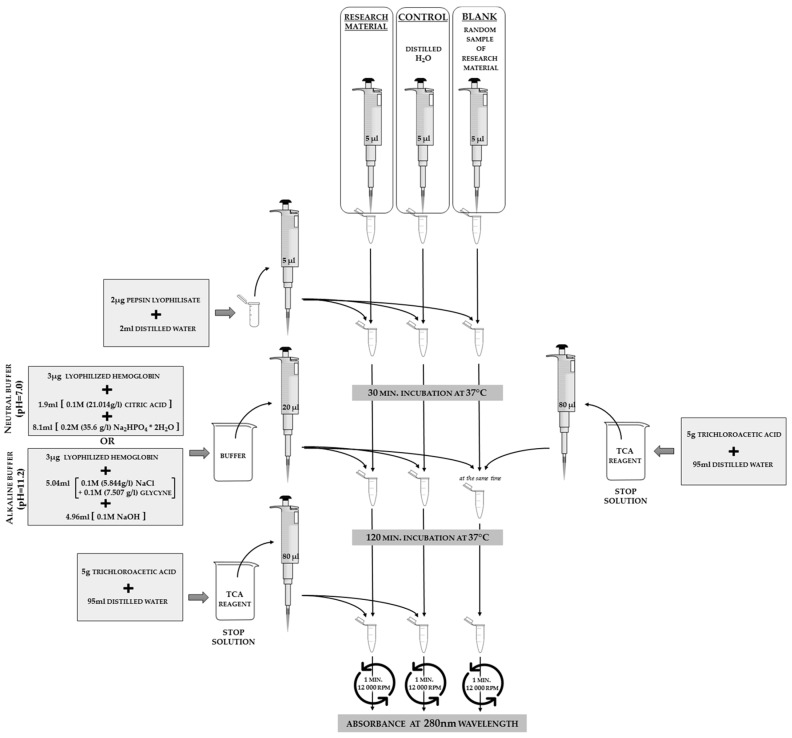
The scheme of neutral (7.0 pH) and alkaline (11.2 pH) protease inhibitor activity analyses in hemolymph solutions or insect body surface elutions. In order to calculate the values of protease inhibitor activities from the absorbance results, it is necessary to substitute the control sample absorbance in the calculation formula in order to determine the results when all the reagents are present but no reaction could occur because there was no biological material. It is also necessary to substitute the blank sample absorbance where the biological material was used, but no reaction occurred (the stop solution is added at the beginning of the procedure, as shown on the right part of the scheme). The phrase “random sample” refers to the case when we analyze a larger number of samples in one study; we may prepare only one control sample and only one blank for all of the samples that use the same set of reagents on the same day.

The levels of protease inhibitors can be determined using the method of Lee and Lin [18] for insects, as adjusted by Strachecka. When determining the general levels of protease inhibitor activities, pepsin is used as a protease marker at an acidic pH, as shown in Figure 8, and trypsin is used as a protease marker at neutral and alkaline pH, as shown in Figure 9.

It takes approximately 3.5 h to determine the protease inhibitor activity analyses for 100 samples and one pH value (this is highly dependable on the laboratory equipment and the spectrophotometer model). The cost of analysis for 100 samples is about $200.

#### 3.2.4. Calculation of Protease Inhibitor Activity Levels

In order to calculate the protease inhibitor activity levels, the absorbance values need to be inserted into the formula, as follows:

(3) $$\text{P}\;\mathrm{ROTEASE}\;\mathrm{INHIBITOR}\;\mathrm{ACTIVITY}=\frac{CONTROL\;ABSORBANCE-SAMPLE\;ABSORBANCE}{CONTROL\;ABSORBANCE\times total\;protein\;concentration\left[\frac{mg}{ml}\right]\;}\left[\frac{U}{ml}\right]$$

where the total protein concentration is the value obtained in the previous analysis for the same sample.

### 3.3. “Liver” Tests

The term “liver” tests is vicarious, because insects have only a liver equivalent: the fat body [7]. Nevertheless, in the hemolymph of insects, there are substances stemming from biochemical transformations that occurred in the fat body. Among others, these include: alanine aminotransferase (ALT), aspartate aminotransferase (AST), and alkaline phosphatase (ALP). The activities of these substances in the tissues of an insect may have a different meaning than in mammals (it depends on the insect species). However, they still can be used to assess the immunity status.

It takes approximetely one hour to determine the ALT, AST, or ALP activity analyses for 100 samples (this is highly dependable on the laboratory equipment and the spectrophotometer model). The cost of analysis for 100 samples is about $50.

#### 3.3.1. Alanine Aminotransferase (ALT)

**Figure 10:**
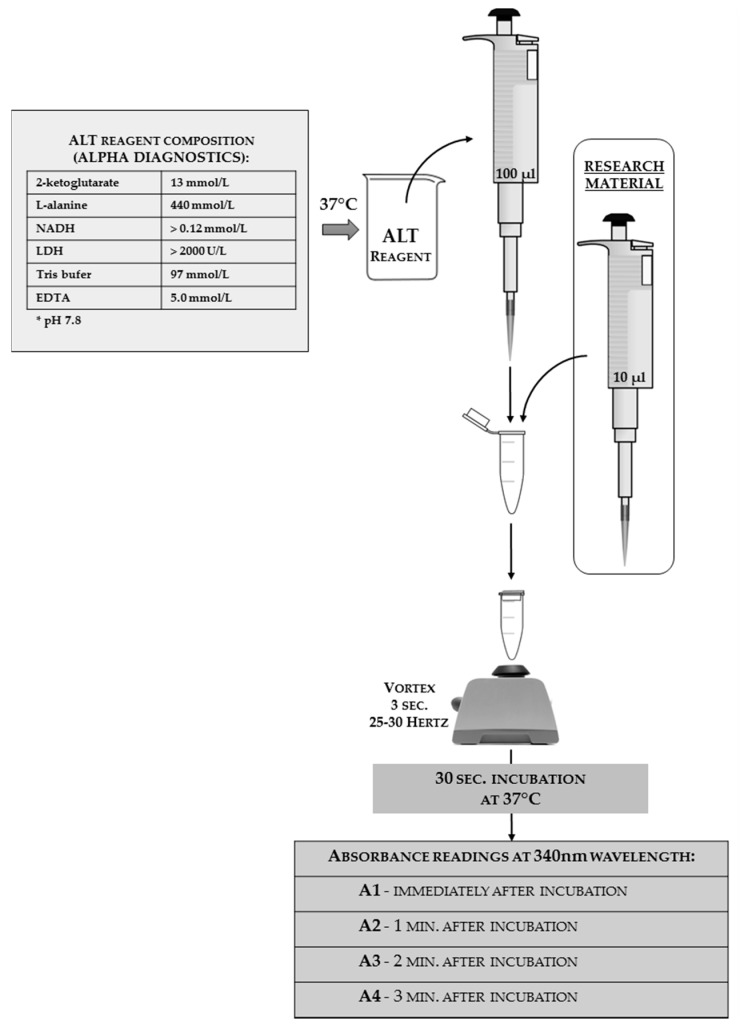
The scheme of alanine aminotransferase (ALT) activity analyses adjusted for insect hemolymph solutions with a one-reagent commercial kit from Alpha Diagnostics^®^. The reagent composition declared by the manufacturer is shown on the left side of the scheme. The applied tools and reagents, along with the used volumes, are presented in the scheme.

The ALT activity analysis can be done using a commercial kit with modified instructions. In Figure 10, the authors present the reaction scheme adjusted for insect hemolymph; the authors used an Alpha Diagnostics^®^ agent as a one-reagent tool (the full reagent composition that was shown on the package is given in the scheme). The same match can be done with products from other companies, or the reagent can be self-made from its chemical components.

#### 3.3.2. Aspartate Aminotransferase (AST)

**Figure 11:**
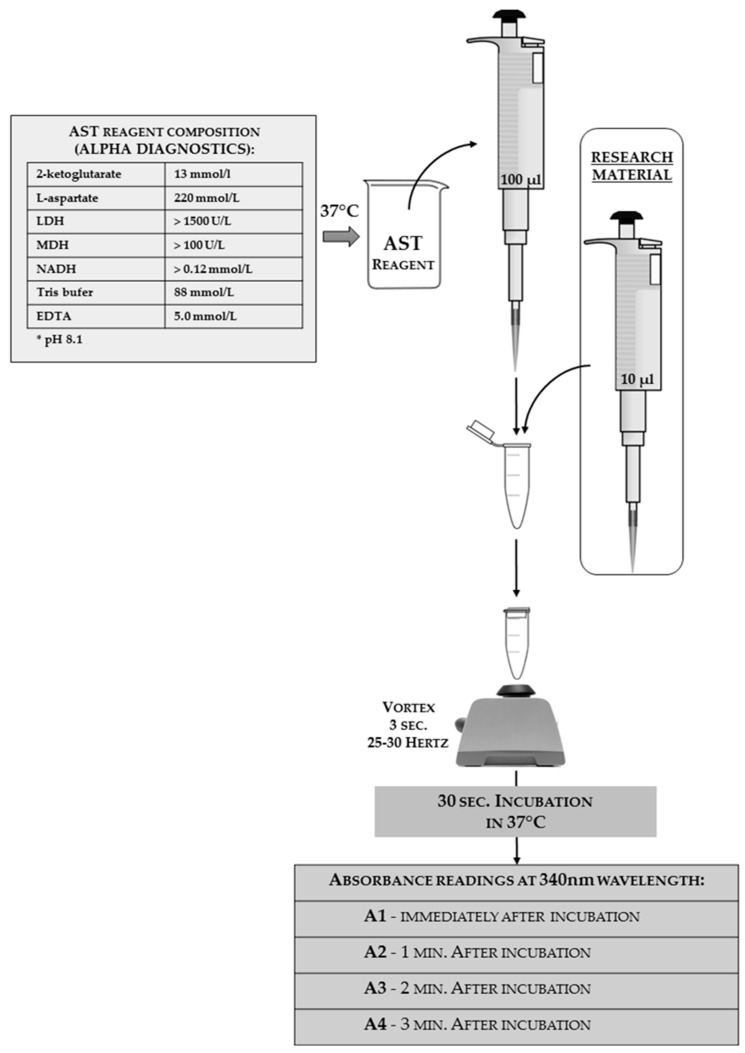
The scheme of aspartate aminotransferase (AST) activity analyses adjusted for insect hemolymph solutions with a one-reagent commercial kit from Alpha Diagnostics^®^. The reagent composition declared by the manufacturer is shown on the left side of the scheme. The applied tools and reagents, along with the used volumes, are presented in the scheme.

The AST activity analysis can be done using a commercial kit with modified instructions. In Figure 11, the authors present the reaction scheme adjusted for insect hemolymph; the authors used an Alpha Diagnostics^®^ agent as a one-reagent tool (the full reagent composition that was shown on the package is given in the scheme). The same match can be done with products from other companies or the reagent can be self-made from its chemical components.

#### 3.3.3. Alkaline Phosphatase (ALP)

**Figure 12:**
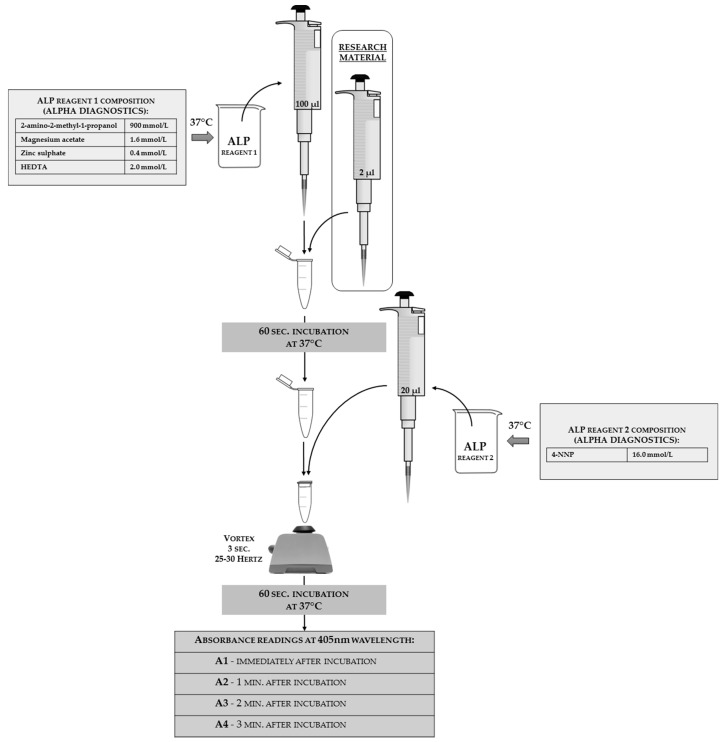
The scheme of alkaline phosphatase (ALP) activity analyses adjusted for insect hemolymph solutions with a two-reagent commercial kit from Alpha Diagnostics^®^. The reagent composition declared by the manufacturer is shown on the left and the right side of the scheme. The applied tools and reagents, along with the used volumes, are presented in the scheme.

The ALP activity analysis can be done using a commercial kit with modified instructions. In Figure 12, the authors present the reaction scheme adjusted for insect hemolymph; the authors used Alpha Diagnostics^®^ agents as a two-reagent tool (the full reagents’ composition that was shown on the package is given in the scheme). The same match can be done with products from other companies or the reagents can be self-made from its chemical components.

#### 3.3.4. Calculation of ALT, AST, and ALP Activities

In order to calculate the activities of ALT, AST, or ALP, the absorbance values need to be inserted into the formula, as follows:

(4) $$ALT\;\mathrm{ACTIVITY}=\frac{\Delta abs}{min}\times FACTOR\left[\frac{U}{l}\right]$$

(5) $$AST\;\mathrm{ACTIVITY}=\frac{\Delta abs}{min}\times FACTOR\left[\frac{U}{l}\right]$$

(6) $$ALP\;\mathrm{ACTIVITY}=\frac{\Delta abs}{min}\times FACTOR\left[\frac{U}{l}\right]$$

(7) $$ALT/AST\;\text{F}\;\mathrm{ACTOR}=\frac{TV\times 1000}{6.3\times SV\times P}$$

(8) $$ALP\;\text{F}\;\mathrm{ACTOR}=\frac{TV\times 1000}{18.8\times SV\times P}$$

(9) $$\frac{\Delta abs}{min}=\frac{\left(A2-A1\right)+\left(A3-A2\right)+\left(A4-A3\right)}{3}$$

where:

A1, A2, A3, A4—individual readings of the absorbance values for the samples
TV—total volume of the reaction mixture
SV—sample volume used for the reaction
P—optical path length of the cuvette
6.3—absorbance factor for dihydronicotinamide adenine dinucleotide (NADH; at 340-nm wavelength)
18.8—absorbance factor for 2,4-dinitrophenol (2,4-DNP)

### 3.4. Urea Concentration

**Figure 13:**
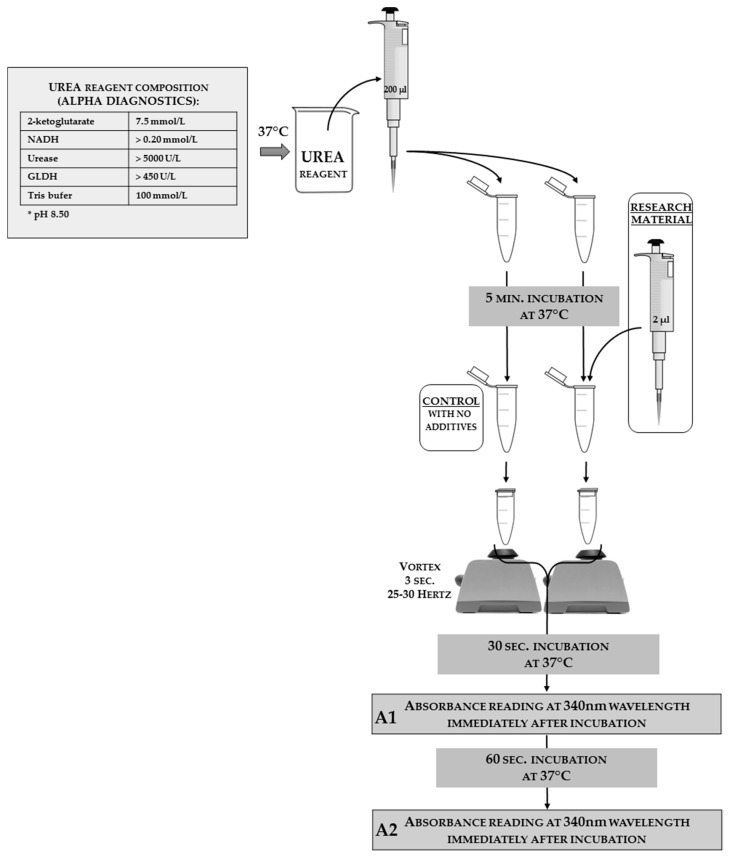
The scheme of urea concentration analysis adjusted for insect hemolymph solutions with a one-reagent commercial kit from Alpha Diagnostics^®^. The reagent composition declared by the manufacturer is shown on the left side of the scheme. The applied tools and reagents, along with the used volumes, are presented in the scheme. In order to calculate the value of the urea concentration from the absorbance results, it is necessary to substitute the control sample absorbance in the calculation formula. This substitution shows the results for when all of the reagents are present, but no reaction could occurs because there was no biological material.

ea concentration in an insect hemolymph solution can be estimated as an additional factor. This analysis can be done using a commercial kit with modified instructions. In Figure 13, the authors present the reaction scheme adjusted for insect hemolymph solutions; the authors used an Alpha Diagnostics^®^ agent as a one-reagent tool (the full reagent composition that was shown on the package is given in the scheme). The same match can be done with products from other companies, or the reagent can be self-made from its chemical components.

It takes approximately one hour to determine the urea concentration analyses for 100 samples (this is highly dependable on the laboratory equipment and the spectrophotometer model). The cost of analysis for 100 samples is about $50.

#### Calculation of the Urea Concentration

In order to calculate the concentration of urea, the absorbance values need to be inserted into the formula, as follows:

(10) $$\begin{array}{c} U\;\mathrm{REA}\;\mathrm{CONCENTRATION}=\frac{SAMPLE\;A2\;-\;SAMPLE\;A1}{CONTROL\;A2\;-\;CONTROL\;A1}\times \\ STANDARD\;CONCENTRATION\left[\frac{mmol}{l}\right]\;or\;\left[\frac{mg}{dl}\right] \end{array}$$

(11) $$\text{S}\;\mathrm{TANDARD}\;\mathrm{CONCENTRATION}=14.3\;\frac{mmol}{l}=40\;\frac{mg}{dl}$$

### 3.5. Glucose Concentration

**Figure 14:**
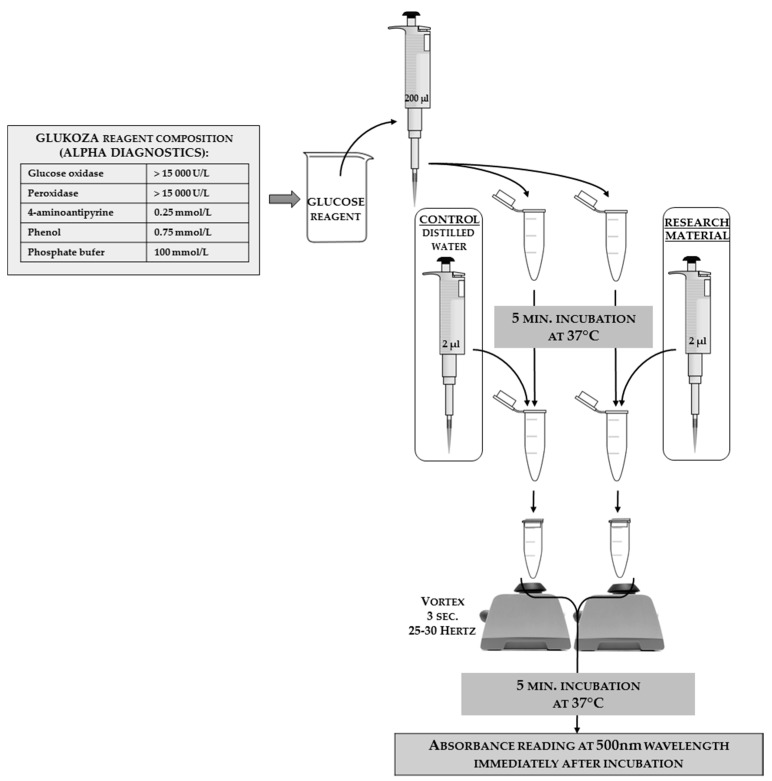
The scheme of glucose concentration analyses adjusted for insect hemolymph solutions with a one-reagent commercial kit from Alpha Diagnostics^®^. The reagent composition declared by the manufacturer is shown on the left side of the scheme. The applied tools and reagents, along with the used volumes, are presented in the scheme. In order to calculate the value of the glucose concentration from the absorbance results, it is necessary to substitute the control sample absorbance in the calculation formula. This substitution shows the results for when all the reagents are present, but no reaction could occur because there was no biological material.

Glucose concentration in insect hemolymph solutions can be estimated as an additional factor. This analysis can be done using a commercial kit with modified instructions. In Figure 14, the authors present the reaction scheme adjusted for insect hemolymph; the authors used an Alpha Diagnostics^®^ agent as a one-reagent tool (the full reagent composition that was shown on the package is given in the scheme). The same match can be done with products from other companies, or the reagent can be self-made from its chemical components.

It takes approximetely one hour to determine the glucose concentration analyses for 100 samples (this is highly dependable on the laboratory equipment and the spectrophotometer model). The cost of analysis for 100 samples is about $50.

#### Calculation of the Glucose Concentration

In order to calculate the concentration of glucose, the absorbance values need to be inserted into the formula as follows:

(12) $$\begin{array}{c} G\;\mathrm{LUCOSE}\;\mathrm{CONCENTRATION}=\frac{SAMPLE\;ABSORBANCE}{CONTROL\;ABSORBANCE}\times \\ STANDARD\;CONCENTRATION\;\left[\frac{mg}{dl}\right] \end{array}$$

$$S\;\mathrm{TANDARD}\;\mathrm{CONCENTRATION}=238\;\frac{mg}{dl}$$

### 3.6. Analysis Adjusted to the Type of Biological Material

Scientists can perform many analyses with biological material such as hemolymph, since it is an equivalent of mammalian blood tissue that distributes all of the substances through the insect body. However, the elution from the surface of the insect body lacks the metabolites that are present in the hemolymph. Therefore, carrying out rapid biochemical tests to determine the activity of the proteolytic system makes sense for the hemolymph solution and the elution from the body surface, which is not the case with rapid “liver” analyses and concentrations of urea and glucose, as shown in Table 3. In addition, due to the correct way of collecting the research biological material, it is possible to perform other types of analysis, e.g., chromatography from both the hemolymph solution and body surface elution, which can verify the presence of health-promoting or harmful substances inside the body and on the body surface.

### 3.7. Additional Tips for Applying Presented Methodologies

**Figure 15:**
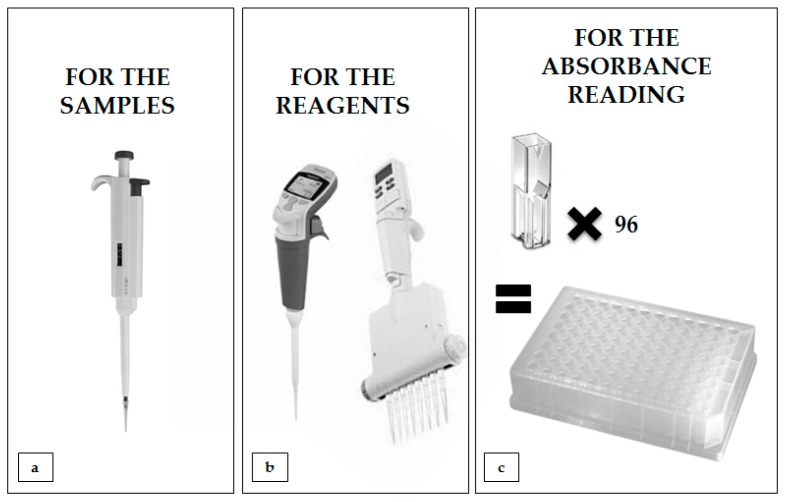
The use of appropriate tools significantly speeds up the biochemical analysis and absorbance reading: (**a**) when dispensing samples with biological material, it is worth using an automatic one-channel pipette with easily changeable tips; (**b**) for pipetting the same volume of the reagent many times, it is worth using an electronic (optionally multi-channel) automatic pipette (there is no need to change the tips until they come in contact with the biological material in the tube); (**c**) if we have a spectrophotometer reading of the absorbance from 96 well plates, we should use it in place of individual cuvettes.

In order for the analyses proposed by the authors to be useful and quick, several aspects of research planning should be taken into account:

- the planned research group should always have an appropriate number of individual insects depending on the type of statistical analysis that the researcher wants to use (the authors suggest not fewer than 10 individual insects per group);
- if possible, it is always worth re-examining or even repeating the experiment to confirm the reliability of the obtained results;
- samples containing the hemolymph solution cannot remain defrosted for a long period of time, because positive temperature and exposition to atmospheric air (and the oxygen contained in it) cause their melanization, which disqualifies them from further analysis;
- in order to carry out all of the analyses as soon as possible, the methods for proteases and protease inhibitors that employ the same buffer in reactions should be performed at the same time;
- during the longer incubations that are required for the analysis of the proteolytic system, carrying out of the rapid “liver” or urea and glucose tests simultaneously is recommended;
- it is worth using modern laboratory equipment to speed up the analysis (e.g., such as in Figure 15);
- in the case of analyses of the proteolytic system, where the penultimate step is sample centrifugation (which is aimed at depositing the unreacted protein precipitate at the bottom of the tube), the entire analysis cannot be carried out on the well plate;
- in the case of “*liver*”, urea, and glucose tests, whole analyses can be conducted on the well plate;
- when analyzing a large number of samples, remember that the reaction begins to occur in the sample from the time the reagent is added; so, the time difference between pipetting the reagents to the first sample and the last sample should be minimal.

## 4. Examples of Result Interpretation

### 4.1. Meanings of the Proteolytic System Activity

The proteolytic system depends on various factors, such as the insects’ age, developmental stage, biostimulators, pathogens, toxins, sex, roles within the caste (in eusocial insects), rearing conditions (temperature, humidity, animal density), and anthropogenic factors, etc. These activities are different for various insect species. The reference values are not estimated. A beneficial factor for one insect species can be negative for another. In order to determine the values of proteolytic system activity and its meaning for an organism, it is necessary to design research that involves an experimental group treated with a factor of known effect on the particular species. The interpretation of results is highly dependable on the characteristics of the species. Therefore, in Section 4, the authors mostly presented interpretations for honey bees that may be valid for other insect species.

#### 4.1.1. The Proteolytic System on the Body Surface

The protein concentration linearly and gradually increases until the 15th to the 18th day on the cuticle of healthy bees; then, it decreases. These age-dependent changes occur in different bees, e.g., *Apis mellifera* or *Osmia rufa* [19]. Unfavorable or harmful factors such as environmental pollution and acaricides contribute to increasing the protein concentration on the body surface of bees [20,21]. This indicates the body’s response to the factor, which includes increased protein synthesis inside the organism and protein secretion on the cuticle of the insect [17].

In healthy bees, the acidic, neutral, and alkaline protease activities increase with the age of bees until the 18th to 20th day; then, they decrease. The activities of neutral protease inhibitors increase during the entire lifetime of the insect, but the activities of the acidic and alkaline protease inhibitors decrease until the 12th to 15th day, and then increase [20]. During contact with unfavorable and harmful factors that are not too strong, the activities of acidic and neutral proteases—as well as acidic and alkaline protease inhibitors—increase until the 14th to the 18th day of insect age; then, they decrease. In this situation, the activities of alkaline proteases and neutral protease inhibitors decrease with the age of the insects [20]. During contact with unfavorable or harmful factors that are very strong, the protease and protease inhibitor activities are lower in comparison to the control group, and linearly/gradually decrease with the age of insects in the treated group [21,22], as shown in Table 4. The imbalance between proteases and their inhibitors on the insect cuticle surfaces that is caused by these factors leads to the weakening of the protective barriers and anatomical–physiological safeguards that result in secondary infection by microorganisms, e.g., fungi or bacteria [21,23]. Moreover, the ratio of compounds that seal the cuticle, such as waxes, esters, hydrocarbons, etc., is changed, and other toxins have easier access to the cuticle and the interior of the insect [24].

#### 4.1.2. The Proteolytic System in the Hemolymph

The protein concentrations increase during the aging/maturation process, but then decrease in older healthy bees. Bees that consumed biostimulators and/or vitamins have higher concentrations of proteins. However, the decrease in older bees begins much later in the experimental group than in the control group [25,26,27]. These substances stimulate and drive protein synthesis in the fat bodies of bees, and are then secreted to the hemolymph. During contact with unfavorable or harmful factors, protein concentrations almost double in the hemolymph of very young bees (2–4 day-old); then, they drastically decrease [20,28]. The first reaction is the mobilization of the whole organism by adverse environmental factors and increased protein synthesis. The organism quickly uses these reserves. Since the fat body is destroyed by strong chemical compounds, it cannot perform its function and produce new proteins that are released into the hemolymph [29].

In healthy bees, acidic and alkaline protease activities increase with age until 20–25 days of age; then, they decrease. The same situation occurs in the case of acidic, neutral, and alkaline protease inhibitors. Neutral protease activities increase with the age of insects [25,27,30]. Bees treated with biostimulators and vitamins have higher activities of proteases and lower activities of protease inhibitors. Ashihara and Crozier [31] reported that substances such as caffeine, coenzyme Q10, and others had long been considered to constitute the chemical defence against biological stressors. Kim and Sano [32] suggest that these substances stimulate the production and/or translocation of protease inhibitors and signaling molecules, and thus enhance resistance against infection by viruses and bacterial pathogens. The enzymes involved in the proteolytic system are essential components of insect resistance barriers, and are associated with the activity of the antioxidant systems. The antioxidative system ensures protection against reactive oxygen species (ROS). Proteins may undergo scission reactions with certain radicals/oxidants, leading to the direct formation of potentially toxic peptide fragments [33,34]. Various intercellular proteolytic enzymes can recognize, and preferentially degrade, oxidatively damaged proteins to small-sized proteins and amino acids. Moreover, proteases and protease inhibitors affect the normal course of the following processes: phagocytosis, melanization, cellular adhesion molecule recognition, the generation of reactive intermediates of oxygen and nitrogen, the activation of proapoptotic molecules, the synthesis of cytokines and antimicrobial peptides, enzyme activation, and molecular and hormonal signaling, as well as pathogen protein degrading [17,35]. During contact with unfavorable or harmful factors, the activities of acidic, neutral, and alkaline proteases increase drastically on the first of the three days, and then decrease (Table 5), and their activities in old bees are lower in the treated group than in the control group. The activities of protease inhibitors are lower in the treated group than in the control group, and they decrease with the age of the insects [20,28]. These changes may adversely affect the abnormal course of the cellular processes, as mentioned above [17,35]. These substances can act directly or indirectly on the activity of the proteolytic system. These compounds destroy cells, change the metabolism of cells and the whole organism, denature proteins, change or block the active centers of enzymes, including the proteolytic ones, block electron transport in the respiratory chain, and thus block ATP synthesis [36,37,38]. Consequently, this weakens the organism, and leads to more frequent infections [23,28].

### 4.2. “Liver” Test Implications

The activities of ALT, AST, and ALP increase during the aging/maturation process in the hemolymph of healthy bees. Following acaricide or antibiotic administration, a decrease is observed in the activities of the enzymatic physiological markers (Table 6) [20,39]. The activities of AST, ALT, and ALP are also reduced by the parasitic activity of *Varroa destructor* [40]. Lower activities of the enzymes in bees may impair the Krebs cycle, ATP synthesis, oxidative phosphorylation, β-oxidation, and other metabolic cycles [41], and may also indicate the presence of low dietary amounts of proteins, which is indispensable for amino acid formation in connection with tissue development, excretion, and energy demand [42]. Moreover, the decreased enzyme activities observed in the hemolymph of honey bees treated with unfavorable or harmful factors could mean that either AST, ALT, or ALP was not released in the treated worker bees (suppression) or that the factors blocked them, thus directly decreasing their activities (inhibition), or both. Bergmeyer et al. [43] suggest that unfavorable or harmful factors binding to AST, ALT, and ALP catalytic centers inhibit the enzyme activities, which also depend on substrate concentrations. Similar effects of activity reduction were observed in the case of diflubenzuron, triflumuron, chlorfluazuron, hexaflumuron, flufenoxuron, and pyriproxyfen treatments against *Spodoptera littoralis* [44]. In some cases, it is difficult to interpret the values of the activities of these “hepatic” enzymes, e.g., the cyromazine treatment against *Culex pipiens* at the concentration of 0.1 ppm caused a decrease in the AST and ALT levels, but the concentration of 1 ppm caused those enzyme activities to increase [45]. In turn, following biostimulator or vitamin administration, an increase is observed in the activities of the enzymatic biomarkers in bees [25,27,30]. In other insects, e.g., *Bombyx mori*, an increase in hemolymph ALT activities in the larvae occurs after the administration of cytotoxic agents. Moreover, the agents cause tissue damage via cytochrome P450 activation and the subsequent production of reactive oxygen species. This ALT increase in *B. mori*, which is caused by carbon tetrachloride, was suppressed by *N*-acetyl-cysteine, a radical scavenger [46,47].

AST, ALT, and ALP are used as indicators of the proper functioning of the liver in mammals. Their increased activities may indicate hepatotoxicity, pathological changes, and chronic diseases. The mechanism of activating these enzymes in bees is opposite to that in mammalians; i.e., harmful factors suppress their activities. This interpretation is only valid for honey bees, and in order to formulate a general principle for all insects (which are the most diverse group of animals, and includes more than one million described species), significantly more analyses are needed.

### 4.3. Urea and Glucose Parameters

Urea concentrations, representing non-enzymatic antioxidants, increase in healthy bees until the 14th day of age; then, they decrease until the death of the workers. Urea concentrations were lower in the biostimulator-treated bees than in the control group [25,27,30]. Urea concentration decreased drastically two to three times on the first days after the administration of strong acaricides [20]. Şapcaliu et al. [48] found that urea concentrations rose in honey bees that were in contact with inconsiderable stress factors (e.g., temperature). This is probably due to the higher intensity of processes related to capturing reactive oxygen forms and binding ions [49]. Moreover, urea is present in the excreta of many insects; however, it usually forms a rather minor component [50].

Glucose concentrations, representing non-enzymatic biomarkers, decrease with the age of healthy workers. Biostimulators increase glucose concentration. During contact with unfavorable or harmful factors, glucose concentrations decrease by almost half in the hemolymph of young and older bees [20,39]. Glucose is one of the compounds involved in energy production processes, which insects use, for example, during the flight. A higher glucose concentration in insects protects them from stress and adverse environmental conditions. Glucose is stored in the fat body in a polymeric form, glycogen, which can be readily degraded on demand to be used as a glycolytic fuel. This compound is the sugar circulating in the hemolymph. Glucose is used for the synthesis of chitin, which is a major cuticle component, and for the synthesis of sugar alcohols, which are needed for adaptation to cold or drought [7,51].

## 5. Conclusions

In summary, in the opinion of the authors, the presented methodologies are a sufficient tool for assessing the first line of insect resistance on the surface of insect body, and a further line that is present in the hemolymph. The results obtained from two types of biological material after carrying out the same (proteolytic system activities) and complementary (“liver”, urea, and glucose tests) analyses allow for an (almost) full definition of the immunological status of the research group. Due to the ease of execution, as well as the speed and cost-effectiveness of the biochemical analyses, they should become a common practice in research that is conducted on various insects species. Different scientists performing the same type of analysis will enable comparisons of insect studies of insects, and make them more universal and ubiquitous.

## Figures and Tables

**Table 1:** Comparison of methods for collecting insect hemolymph (“blood”).

	**Collecting Hemolymph:**		
	flowing out after detachment of the antennae	flowing out after incision of the body with a scalpel	into a glass capillary by puncturing the body
description of the sampling technique	1. Holding the insect thorax and head immobilization (e.g., with fingers or with tweezers). ^1^ 2. Removing the antennae with tweezers. 3. Collecting floating hemolymph from the head surface with a pipette. ^2^ * scheme in Figure 1	1. Insect immobilization (e.g., with fingers or with tweezers). ^1^ 2. Incision of body layers with a scalpel. ^1^ 3. Collecting floating hemolymph with a pipette. * scheme in Figure 2	1. Holding and immobilization of the insect with tweezers. ^1^ 2. Puncturing the abdomen with a sterile preparation needle. 3. Placing a glass capillary inside the abdomen and collecting hemolymph straight from the venous sinus. ^3^ * scheme in Figure 3 * demonstrative photo in Figure 4
difficulty level	easy	difficult	difficult and requiring precision
insects on which the method can be used	adult imago with antennae	insects with non-chitinized body surface	insects with developed venous sinuses
need for special tools	disposable medical gloves tweezers pipette pipette tips beaker with 0.6% NaCl Eppendorf tube	disposable medical gloves tweezers pipette pipette tips beaker with 0.6% NaCl scalpel Eppendorf tube	disposable medical gloves tweezers preparation needle glass capillary pipette pipette tips beaker with 0.6% NaCl micropipette ^2^ Eppendorf tube
microbiological contamination of the collected material	likely	very likely	unlikely
amount of collected material (e.g., from two-day-old honey bees) (*personal observation*)	about 5–10 µL	about 2–8 µL	about 6–20 µL
storage conditions	Hemolymph suspended in physiological saline in the Eppendorf tube can be frozen at −20 °C for about three months before enzymes and proteins lose activity.	Hemolymph suspended in physiological saline in the Eppendorf tube can be frozen at −20 °C for about three months before enzymes and proteins lose activity.	Hemolymph inside the glass capillary placed in physiological saline in the Eppendorf tube can be frozen at −20 °C for about eight months before enzymes and proteins lose activity, or hemolymph blown out from the glass capillary and suspended in physiological saline in the Eppendorf tube can be frozen at −20 °C for about three months before enzymes and proteins lose activity.

^1^ The applied technique of insect immobilization is dependent on the development stage and species of the insect; the authors do not recommend using chemical or physical methods of immobilization, because they affect the results. ^2^ Possibility of biological material contamination. ^3^ The use of a micropipette is necessary in this technique if we want to blow out the hemolymph from the inside of the glass capillary; unless we do this, concentrated, pure hemolymph is trapped inside the glass capillary by means of cohesion and adhesion forces.

**Table 2:** Preparation of the required pH value from two stock solutions (A and B).

pH	Composition of the Buffer Solution	
	A (mL)	B (mL)
	0.1 M (21.014 g/L) Citric Acid Monohydrate	0.2 M (35.60 g/L) Na_2_HPO_4_·2H_2_O
**2.2**	9.88	0.12
**2.4**	9.45	0.55
**2.6**	9.00	1.00
**2.8**	8.51	1.49
**3.0**	8.03	1.97
**3.2**	7.60	2.40
**3.4**	7.20	2.80
**3.6**	6.84	3.16
**3.8**	6.51	3.49
**4.0**	6.20	3.80
**4.2**	5.91	4.09
**4.4**	5.64	4.36
**4.6**	5.37	4.63
**4.8**	5.12	4.88
**5.0**	4.90	5.10
**5.2**	4.69	5.31
**5.4**	4.47	5.53
**5.6**	4.22	5.78
**5.8**	4.00	6.00
**6.0**	3.74	6.26
**6.2**	3.45	6.55
**6.4**	3.14	6.86
**6.6**	2.79	7.21
**6.8**	2.35	7.65
**7.0**	1.90	8.10
**7.2**	1.38	8.62
**7.4**	0.98	9.02
**7.6**	0.68	9.32
**7.8**	0.46	9.54
	**0.1 M (20.62 g/L)** **5,5-Diethylbarbituric Acid Sodium Salt**	**0.1 M** **HCL**
**8.0**	7.06	2.94
**8.2**	7.56	2.44
**8.4**	8.12	1.88
	**0.1M (7.507 g/L) Glycine** **+0.1M (5.844 g/L) NaCL**	**0.1 M** **NaOH**
**8.6**	9.47	0.53
**8.8**	9.20	0.80
**9.0**	8.84	1.16
**9.2**	8.40	1.60
**9.4**	7.89	2.11
**9.6**	7.32	2.68
**9.8**	6.72	3.28
**10.0**	6.25	3.75
**10.2**	5.88	4.12
**10.4**	5.57	4.43
**10.6**	5.36	4.64
**10.8**	5.22	4.78
**11.0**	5.12	4.88
**11.2**	5.04	4.96
**11.4**	4.95	5.05
**11.6**	4.87	5.13
**11.8**	4.76	5.24
**12.0**	4.60	5.40
**12.2**	4.32	5.68
**12.4**	3.91	6.09
**12.6**	3.18	6.82
**12.8**	2.14	7.86

**Table 3:** Comparison of methodologies of rapid biochemical analyses that are legitimate to use on biological materials obtained from insects.

Type of Analyses		Hemolymph Solution	Body Surface Elution
total protein concentration		+	+
Proteases activity	acidic	+	+
	neutral	+	+
	alkaline	+	+
proteases inhibitors activity	acidic	+	+
	neutral	+	+
	alkaline	+	+
“*liver*” tests	ALT	+	−
	AST	+	−
	ALP	+	−
urea concentration		+	−
glucose concentration		+	−

**Table 4:** Simplified interpretation of the proteolytic system activity results from the honey bee body surface elutions during contact with unfavorable or harmful factors.

		The Sample Result Compared with the Control Group Sample Result Is:	
		Increased	Decreased
**Protease activity**	**acidic**	−	+
	**neutral**	−	+
	**alkaline**	−	+
**Protease inhibitor activity**	**acidic**	−	+
	**neutral**	−	+
	**alkaline**	−	+

**Table 5:** Simplified interpretation of the proteolytic system activity results from the honey bee hemolymph solutions during contact with unfavorable/harmful factors.

		The Sample Result Compared with the Control Group Sample Result is:	
		Increased	Decreased
**Protease activity**	**acidic**	“+” only on the first of the three days, and “−“ later on	+
	**neutral**		+
	**alkaline**		+
**Protease inhibitor activity**	**acidic**	−	+
	**neutral**	−	+
	**alkaline**	−	+

**Table 6:** Simplified interpretation of the “liver” test results from honey bee hemolymph solutions.

	**The Sample Result Compared to the Control Group Sample Result Is:**	
	**Increased**	**Decreased**
**ALT**	-with age; -during biostimulator or vitamin administration	-after contact with unfavorable/ harmful factors
**AST**		
**ALP**		
